# Combination of inflammatory score/liver function and AFP improves the diagnostic accuracy of HBV‐related hepatocellular carcinoma

**DOI:** 10.1002/cam4.2968

**Published:** 2020-03-09

**Authors:** Yezhou Ding, Kehui Liu, Yumin Xu, Qingqing Zhao, Shike Lou, Xiaogang Xiang, Lei Yan, Zhujun Cao, Qing Xie, Chuanwu Zhu, Shisan Bao, Hui Wang

**Affiliations:** ^1^ Department of Infectious Diseases and Hepatology Ruijin Hospital Shanghai Jiao Tong University School of Medicine Shanghai China; ^2^ Department of Infectious Diseases Ruijin Hospital North Shanghai Jiao Tong University School of Medicine Shanghai China; ^3^ Department of Infectious Diseases The Fifth People's Hospital of Suzhou Jiangsu China; ^4^ Discipline of Pathology School of Medical Sciences and Bosch Institute Charles Perkin Centre The University of Sydney New South Wales Australia

**Keywords:** alanine aminotransferase, alpha‐fetoprotein, aspartate aminotransferase, hepatocellular carcinoma, neutrophil‐lymphocyte ratio

## Abstract

**Background:**

Alpha‐fetoprotein (AFP), routinely used for diagnosis of hepatocellular carcinoma (HCC), is limited with relatively low sensitivity and high false positivity in HBV‐related HCC (HBV‐HCC). Thus, an alternative approach was explored to improve specificity/sensitivity for diagnosis of HBV‐HCC, using the combination of AFP, inflammatory score, and liver function.

**Methods:**

Chronic hepatitis B (CHB) (n = 510) and HBV‐HCC (n = 473) patients were identified retrospectively for this study. The diagnostic value of single vs combined biomarkers for HBV‐HCC was analyzed, using ROC curve.

**Results:**

It was observed that elderliness, male sex, cirrhosis, HBeAg^+^ or no‐antiviral therapy, and elevation of ALT, AST, neutrophil‐lymphocyte ratio (NLR), and AFP were associated with developing HBV‐HCC. However, the cut‐off ALT defined by Chinese standard, but not by AASLD, was a risk factor. Interestingly, AFP of HBeAg^‐^ HBV‐HCC patients without cirrhosis was significantly higher than that of the HBeAg^+^ patients. AUC values for AFP, ALT, AST, or NLR were 0.84 (95% CI: 0.815*‐*0.862), 0.533 (95% CI: 0.501*‐*0.565), 0.696 (95% CI: 0.666*‐*0.725), or 0.684 (95% CI: 0.654*‐*0.713) with optimal cut‐off at 7.21 ng/mL, 43 IU/mL, 38 IU/mL, or 2.61, respectively. Combination of AFP with ALT, AST, and NLR improved the diagnostic performance for HBV‐HCC, compared to any of the single biomarkers or any other combinations among these patients (except no‐cirrhosis).

**Conclusions:**

Elderliness, male sex, elevated ALT, AST, NLR, AFP, cirrhosis, HBeAg^+^, and no‐antiviral treatment were independent risk factors for HBV‐HCC. AASLD standard of ALT cut‐off value may not be suitable for the Chinese population. Regular monitoring of HCC among HBeAg^‐^ patients with abnormal AFP may improve the management of HBV‐HCC. The diagnostic performance of AFP combined with ALT, AST, and NLR for HBV‐HCC was superior to single biomarker or any other combinations among these patients, and its diagnostic equation can be used as useful tool for differentiation of HBV‐HCC from CHB.

## Graphical abstract

The major HBV‐HCC Chinese patients had poor prognosis due to late diagnosis. Improved detecting HBV‐HCC at early stage may improve the prognosis of patients. AFP displays relatively low sensitivity and high false‐positivity in diagnosis of HCC. The diagnostic performance of AFP plus ALT, AST, NLR in HBV‐HCC was significantly better than single or any other combinations. Such finding might be useful in improving accuracy of diagnosis for HBV‐HCC.

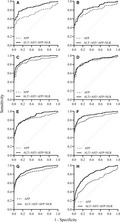

## 1 INTRODUCTION

Hepatocellular carcinoma (HCC) accounts for nearly 85% of liver cancers with a poor prognosis,^1^, ^2^ mainly due to the lack of specific symptoms and/or signs at the early stages of HCC.^3^ The majority of HCC is related to the underlying liver diseases, particularly hepatitis B virus (HBV) infection in China.^3^, ^4^ Therefore, it is critical to improve the detection of HCC at an early stage with reliable sensitivity and specificity, which could have a significant impact on the management of HBV‐HCC.

Serum tumor biomarkers are routinely used for surveillance and diagnosis of HCC, because of their noninvasive nature with relative objective and reproducible quantification.^5^ Serum alpha‐fetoprotein (AFP), probably the most commonly used for the auxiliary diagnosis of HCC, is associated with HCC size, differentiation, invasion, and metastasis.^6^ Serum AFP, however, is sometimes not entirely trustworthy, because AFP is often below the diagnostic value in some early‐stage or even a few late‐stage HCC cases.^7^ In contrast, AFP is elevated substantially in some patients with chronic hepatitis B (CHB), liver cirrhosis, and benign hepatic tumor diseases.^6^, ^7^ Thus, AFP is a biomarker for reference only in the early diagnosis HCC without other supporting clinical information.

To overcome this problem, imaging techniques are being developed, such as computed tomography (CT) or magnetic resonance imaging (MRI), in an attempt to enhance both sensitivity and specificity in the accuracy of early diagnosis.^6^, ^8^ However, the main limitation of using CT and/or MRI is its high cost, as well as, a relative lack of sufficient competent technicians and specialists, which is more of a challenge in rural regions without adequate support.^7^, ^9^

It is well known that the progression of HBV‐HCC is closely associated with inflammatory response and immune status of the microenvironment.^10^ It has been demonstrated that neutrophil‐lymphocyte ratio (NLR) and platelet‐lymphocyte ratio (PLR) are associated with the prognosis of cancers in the lung, colorectum, stomach, and esophagus.^11^, ^12^ Systemic immune‐inflammation index (SII) is another parameter that integrates lymphocytes, neutrophils, and platelets for evaluating inflammation objectively.^13^ Both NLR and PLR are good in predicting the prognosis of lung, colorectum, stomach, esophagus, and HCC in a small cohort.^14^ Although SII is considered to be an independent prognostic predictor for nonsmall cell lung cancer (NSCLC) and HCC,^13^ it is unknown whether the combination of AFP, NLR, PLR, and SII could improve the sensitivity and specificity in the diagnosis and/or prediction of HBV‐HCC.

The aim of the current study was to explore the diagnostic value of SII, NLR, and PLR in HBV‐HCC patients, and to further evaluate if the combination of SII, NLR, and PLR with AFP during the development of HBV‐HCC could improve the diagnostic value. Alanine aminotransferase (ALT) and aspartate aminotransferase (AST), both classic routinely used clinical serum biomarkers for determination of liver function were also included as references to the incidence of HBV‐HCC.

## 2 MATERIAL AND METHODS

### 2.1 Study design

**Figure 1:**
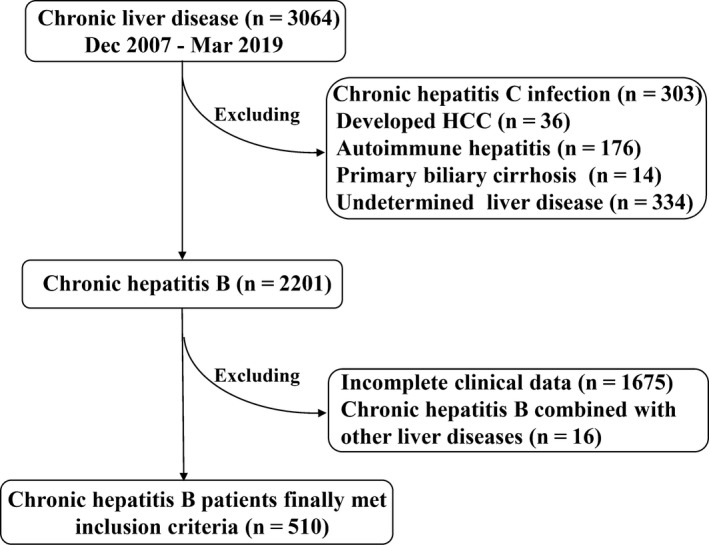
CHB Patients’ selection procedures

CHB and HBV‐HCC patients were identified in the Ruijin Hospital (Shanghai, China) from December 2007 and March 2019. The definition of CHB was based on *American Association for the Study of Liver diseases* (AASLD) 2018 Hepatitis B Guidance, that is,^15^ the persistent presence of hepatitis B surface antigen (HBsAg) for more than 6 months. The exclusion criteria of CHB for the current study were as follows: hepatitis C virus (HCV) infection (n = 303), autoimmune hepatitis (AIH, n = 176), primary biliary cholangitis (PBC, n = 14), HCC (n = 36), and undetermined liver disease (n = 334). The total number of chronic liver diseases identified originally was 3064, but only 2201 CHB patients remained after exclusion based on the above‐mentioned criteria. Furthermore, the patients without complete data (n = 1675) or who had other liver diseases (n = 16) were excluded. Thus a total of 510 CHB patients remained in the current study (Figure 1).

**Figure 2:**
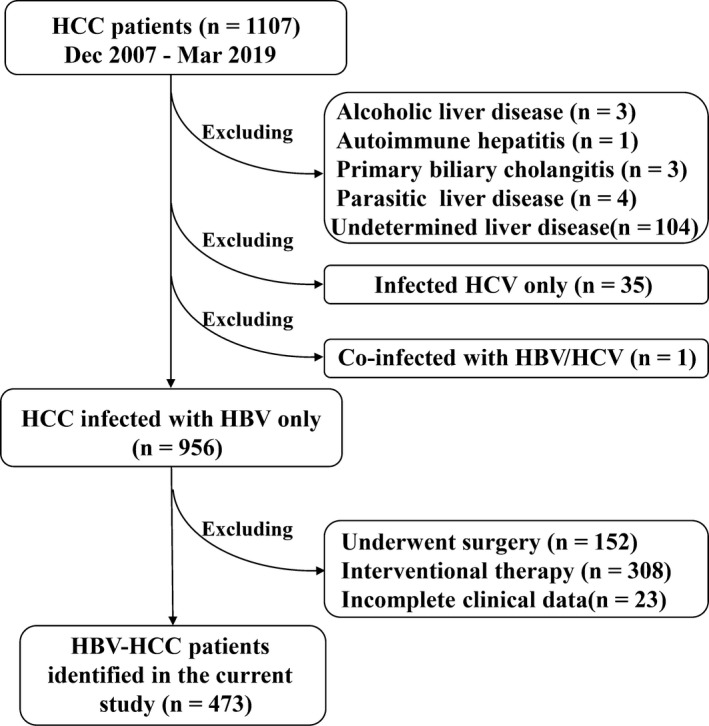
HBV‐HCC Patients’ selection procedures

In this study, the eligible HCC patients (n = 1107) (including both HBV and other causes) who were treatment‐naïve and complete clinical characteristic and laboratory data at the time of diagnosis of HCC, based on AASLD guidelines for the treatment of HCC.^16^ Moreover, patients (n = 151) with the following causes for the development of HCC were excluded for the current study: alcoholic liver disease (n = 3), AIH (n = 1), PBC (n = 3), parasitic liver disease (n = 4), undetermined liver diseases (n = 104), HCV infection (n = 35), HBV/HCV coinfection (n = 1), patients who received surgery (n = 152), intervention therapy (n = 308), or patients who had incomplete data (n = 23). The finally eligible CHB‐related HCC patients were 473 (Figure 2).

### 2.2 Data collection

Serum AST, ALT, and AFP, the numbers of neutrophils lymphocytes and platelets, and virologic examination results were collected from the clinical archived data base. SII = platelet count × neutrophil/lymphocyte count; NLR = neutrophil‐lymphocyte ratio, and PLR = platelet‐lymphocyte ratio. The normal cut‐off ALT was defined as such: ALT < 64 IU/L, AST < 40 IU/L, AFP < 8.78 ng/mL, based on The Chinese National criteria.^17^ According to AASLD 2018 Hepatitis B Guidance, the normal cut‐off was as such: ALT < 35 U/L for males and ALT < 25 U/L for females. The normal cut‐off for SII < 303.9/≥303.9, NLR < 2.07/≥2.07, or PLR < 97.5/≥97.5, respectively. Liver cirrhosis was confirmed based on the results of imaging examinations such as ultrasound or CT or MRI.

### 2.3 Statistical analysis

All statistical analyses were performed using SPSS version 22.0 software (SPSS Inc, Chicago, IL, USA). Data were presented as mean ± standard derivation for normally distributed continuous data, as median (interquartile range, Q25‐Q75) for abnormally distributed continuous data, or as actual values for categorical data. Baseline characteristics were summarized, using descriptive statistics. Groups were compared using χ^2^ tests for categorical, Mann‐Whitney U tests for continuous variables and one‐way ANOVA tests for compare three or more independent groups. Binary logistic regression analysis was applied to determine the best equation for probability prediction of HBV‐HCC from CHB. Receiver operation characteristic (ROC) curves were used to compare the diagnostic performance for each biomarker. The area under the ROC curve (AUC) of each biomarker for distinguishing HBV‐HCC and CHB patients, as well as, the optimal cut‐off value, sensitivity, specificity, positive likelihood ratio (LR+), negative likelihood ratio (LR‐), 95% confidence interval (95% CI) were calculated by MedCalc statistical software. Combinations of markers and other parameters were analyzed, by SPSS 22. A value of *P* < .05 was considered to indicate a statistically significant difference.

## 3 RESULTS

### 3.1 Clinical characteristics of the CHB and HBV‐HCC participants

**Figure 3:**
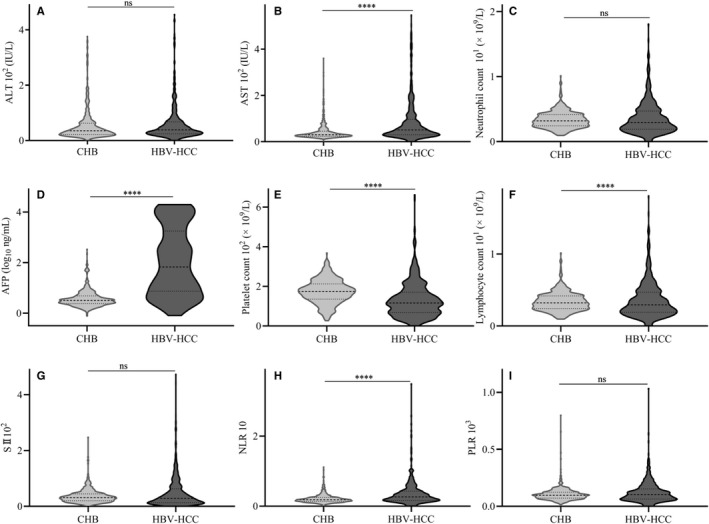
Distribution of serum biomarkers value in CHB and HBV‐HCC group

Among a total of 983 patients, 510 or 473 were CHB or HBV‐HCC in this study, respectively. The clinical characteristics of all of these patients are shown in Table 1. The average age of CHB or HBV‐HCC patients was 39.9 or 55.33, respectively. Male vs female in CHB or HBV‐HCC group were 336 vs 174 or 409 vs 64, respectively. The average level of ALT was 35 or 38 IU/L in CHB or HBV‐HCC groups; while the average level of AST was 30 or 52 IU/L in these two groups, respectively. The average level of AFP, NLR, PLR from the HBV‐HCC group was significantly higher than the CHB group. However there was no significant difference of SII between CHB and HBV‐HCC groups (Figure 3).

**Table 1:** Clinical characteristics

Parameter	Total (N = 983)	Chronic hepatitis B (n = 510)	HBV‐HCC (n = 473)	*P* value
Age (years)	47.33 ± 13.69	39.9 ± 11.78	55.33 ± 10.8	<.001
Sex: Male	745 (75.79%)	336 (65.88%)	409 (86.47%)	<.001
Sex: Female	238 (24.21%)	174 (34.12%)	64 (13.53%)	
ALT (IU/L)	37 (24‐64.5)	35 (22‐64)	38 (25‐67)	NS
AST (IU/L)	37 (26‐70)	30 (24‐46.25)	52 (32‐107)	<.001
Neutrophil count (×10^9^/L)	3.15 (2.2‐4.3)	3.2 (2.42‐4.17)	3 (1.9‐4.7)	NS
Lymphocyte count (×10^9^/L)	1.5 (1‐1.98)	1.8 (1.4‐2.2)	1.14 (0.8‐1.5)	<.001
Platelet count (×10^9^/L)	149 (100‐199)	173.5 (135.5‐212)	116 (69‐170)	<.001
AFP (ng/mL)	4.91 (2.71‐60.2)	3.2 (2.37‐4.8)	64.45 (6.9‐1713)	<.001
SII	303.9 (171‐485.6)	309.6 (200.9‐445.2)	288 (133.9‐638.6)	NS
NLR	2.07 (1.48‐3.28)	1.82 (1.35‐2.44)	2.66 (1.71‐4.3)	<.001
PLR	97.5 (68.44‐133.2)	96.35 (72.17‐123.6)	103.2 (64.37‐151)	NS
Cirrhosis	427 (43.44%)	50 (9.8%)	377 (79.7%)	<.001
HBeAg negative	370 (37.64%)	246 (48.24%)	124 (26.22%)	<.001
HBeAg positive	613 (62.36%)	264 (51.76%)	349 (73.78%)	
With antiviral therapy	459 (46.69%)	252 (49.41%)	207 (43.76%)	NS
HBV DNA < 2000 IU/mL	564 (57.38%)	276 (54.12%)	288 (60.89%)	<.05
HBV DNA ≥ 2000 IU/mL	419 (42.62%)	234 (45.89%)	185 (39.11%)	

Abbreviations AFP, alpha‐fetoprotein; ALT, alanine aminotransferase; AST, aspartate aminotransferase; CHB, chronic hepatitis B; HCC, hepatocellular carcinoma; NLR, neutrophil‐lymphocyte ratio; PLR, platelet‐lymphocyte ratio; HBeAg, hepatitis B e antigen; SII, systemic immune‐inflammation index.

Among CHB or HBV‐HCC groups, 50 (~10%) of 510 or 377 (~80%) of 473 had developed cirrhosis at the time of CHB or HBV‐HCC diagnosed, respectively. In addition, 264 (>51%) or 349 (>73%) of patients were HBeAg^+^ in CHB or HBV‐HCC groups. Among CHB or HBV‐HCC patients, only 252 (~50%) or 207 (>43%) patients received antiviral treatment, including nucleos(t)ide analogs (NAs) or interferon therapy or NAs plus interferon therapy, respectively. Finally, 234 (>45%) or 185 (~40%) of 510 CHB or 473 HBV‐HCC patients, respectively, had HBV DNA ≥2000 IU/mL at the time of CHB or HCC diagnosis (Table 1).

### 3.2 Key parameters related to HBV‐HCC in this study

In order to identify the key risk factor(s) for the onset of HBV‐HCC, the univariate analysis was applied and showed as follows: age (odds ratio, 1.114 [95% CI, 1.099‐1.13], *P* < .05), sex (3.309 [95% CI: 2.401‐4.561], *P* < .05), serum ALT ≥ 64 IU/L (2.145 [95% CI: 1.639‐2.806], *P* < .05), serum AST ≥ 40 IU/L (3.504 [95% CI: 2.693‐4.56], *P* < .05), AFP ≥ 8.78 ng/mL (24.238 [95% CI: 16.811‐34.945], *P* < .05), SII ≥ 303.9 (1.077 [95% CI: 0.837‐1.385], *P* = .563), NLR ≥ 2.07 (0.34 [95% CI: 0.263‐0.443], *P* < .05), PLR ≥ 97.5 (0.872 [95% CI: 0.678‐1.122], *P* = .288), cirrhosis (36.129[95% CI: 25.007‐52.198], *P* < .05), HBeAg^+^ (3.02 [95% CI: 2.309‐3.951], *P* < .05), and with antiviral (0.804 [95% CI: 0.625‐1.033], *P* = .088) and HBV DNA ≥ 2000 IU/mL (1.309 [95% CI: 1.016‐1.688], *P* < .05) (Table 2).

**Table 2:** Univariate and multivariate analysis of risk factors associated with HBV‐HCC

Parameter	Univariate analysis OR (95% CI)	*P* value	Multivariate analysis OR (95% CI)	*P* value
Age	1.114 (1.099‐1.13)	<.05	1.103 (1.077‐1.129)	<.05
Sex: male/female	3.309 (2.401‐4.561)	<.05	2.547 (1.387‐4.676)	<.05
ALT: <64/≥64 IU/L	2.145 (1.639‐2.806)	<.05	11.997 (5.074‐29.665)	<.05
AST: <40/≥40 IU/L	3.504 (2.693‐4.56)	<.05	4.901 (2.204‐10.899)	<.05
ALT: <35/≥35 U/L (M), <25/≥25 U/L(F)	1.008 (0.783‐1.297)	NS		NA
AFP: <8.78/≥8.78 ng/mL	24.238 (16.811‐34.945)	<.05	23.552 (12.275‐45.19)	<.05
SII: <303.9/≥303.9	1.077 (0.837‐1.385)	NS		NA
NLR: <2.07/≥2.07	0.341 (0.263‐0.443)	<.05	0.424 (0.253‐0.709)	<.001
PLR: <97.5/≥97.5	0.872 (0.678‐1.122)	NS		NA
Cirrhosis: No/Yes	36.129 (25.007‐52.198)	<.05	19.046 (10.763‐33.705)	<.05
HBeAg negative/positive	3.02 (2.309‐3.951)	<.05	2.004 (1.13‐3.554)	<.05
Without/with antiviral therapy	0.804 (0.625‐1.033)	NS	0.407 (0.234‐0.708)	<.001
HBV DNA: <2000/≥2000 IU/mL	1.309 (1.016‐1.688)	<.05		NA

Abbreviations AFP, alpha‐fetoprotein; ALT, alanine aminotransferase; AST, aspartate aminotransferase; NLR, neutrophil‐lymphocyte ratio; PLR, platelet‐lymphocyte ratio, HBeAg, hepatitis B e antigen.; SII, systemic immune‐inflammation index.

To minimize the interference from other factors, multivariate analysis was also applied to identify the significant factors. It was observed that age (1.103 [95% CI: 1.077‐1.129], *P* < .05), sex (2.547 [95% CI: 1.387‐4.676], *P* < .05), serum ALT ≥ 64 IU/L (11.997 [95% CI: 5.074‐29.665], *P* < .05), serum AST ≥ 40 IU/L (4.901 [95% CI: 2.204‐10.899], *P* < .05), AFP ≥ 8.78ng/mL (23.552 [95% CI: 12.275‐45.19], *P* < .05), NLR ≥ 2.07 (0.424 [95% CI: 0.253‐0.709], *P* < .001), cirrhosis (19.046 [95% CI: 10.763‐33.705], *P* < .05), and HBeAg^+^(2.004 [95% CI: 1.13‐3.554], *P* < .05), with antiviral (0.407 [95% CI: 0.234‐0.708], *P* < .001) were significantly correlated with HBV‐HCC (Table 2). Interestingly, ALT was not considered as a risk factor for the development of HBV‐HCC among these Chinese patients if the AASLD standard was applied, using either univariate or multivariate analysis.

Finally, elderliness, male, cirrhosis, HBeAg^+^, or no‐antiviral therapy, and elevation of ALT, AST, neutrophil‐lymphocyte ratio (NLR), and AFP were all be independent predictors of HBV‐HCC occurring in these CHB patients (Table 2).

### 3.3 Baseline characteristics of each subgroup biomarkers in this study

Based on the univariate and multivariate analysis, these CHB and HBV‐HCC patients were divided into six subgroups: cirrhosis, without cirrhosis, HBeAg^+^, HBeAg^‐^, with, and without antiviral treatment.

**Figure 4:**
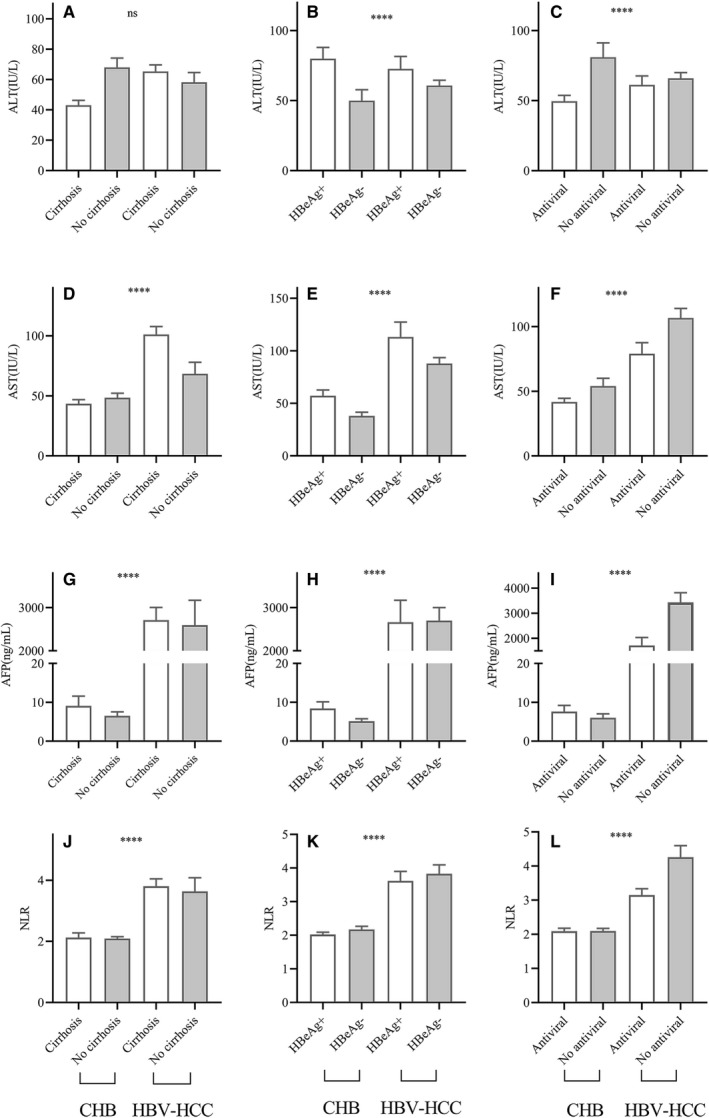
Comparison of ALT, AST, AFP, and NLR levels in CHB and HBV‐HCC patients

After comparing the levels of ALT, AST, AFP, and NLR in patients, respectively, we found that HBeAg^+^ and antiviral therapy had significant effects on ALT levels (*P* < .0001) (Figure 4A‐C), while cirrhosis, HBeAg^+^ and antiviral treatment had significant effects on AST, AFP, and NLR (*P* < .0001) (Figure 4D‐L). In CHB patients, the level of ALT was ~1.6‐fold higher in the HBeAg^+^ group than that in the HBeAg^‐^ group (*P* < .0001), and the level of ALT was ~1.2‐fold lower in the CHB with antiviral treatment compared to that in the group without antiviral (*P* < .01). Among HBV‐HCC patients, ALT in the HBeAg^‐^ group and antiviral group was 14% lower than that in the antiviral group and HBeAg^+^ group (*P* < .05) (Figure 4B,C). Serum AST levels were ~1.3‐fold higher in the HBeAg^+^ group than that in the HBeAg^‐^ group among CHB patients (*P* < .0001), and the AST levels of the with cirrhosis group or the HBeAg^+^ group in HBV‐HCC patients were ~ 1.4‐fold or ~1.5‐fold higher than those in the group of without cirrhosis (*P* < .001) and HBeAg^‐^ group (*P* < .05), while the AST level of the antiviral group, was still 30% lower than that in the group without antiviral (*P* < .05) (Figure 4D‐F). In addition to 1.2‐fold increasing AFP in HBV patients with cirrhosis compare to CHB patients without cirrhosis (*P* < .05), the level of AFP in HBV‐HCC patients with antiviral therapy was ~60% lower than those in the patients without no‐antiviral (*P* < .05). Moreover, there was no significant decrease in the AFP levels of CHB‐HCC patients with HBeAg^‐^ or with cirrhosis compared to patients with HBeAg^+^ or with cirrhosis (*P* > .05) (Figure 4G‐I). Although NLR has no significant difference in each subgroup of CHB (*P* > .05), antiviral therapy in HBV‐HCC patients reduce >19% NLR scores (*P* < .001) (Figure 4J‐L).

Among HBeAg^+^ CHB patients, the AFP of the cirrhotic group was ~1.2‐fold higher than those in the without cirrhotic group (*P* < .05) (Figure S1E‐H). Among CHB patients with or without antiviral, AFP of cirrhotic group was 1.2‐ or 1.7‐fold higher than no‐cirrhotic group (*P < .*05) or (*P* = .01), but there was no significant difference in AFP between HBeAg^‐^ and HBeAg^+^ groups (Figure S1I‐L).

Among HBV‐HCC patients (Figure S2), AFP of antiviral group was 68% lower than no‐antiviral group in patients with cirrhosis (*P* = .005). Although there was no significant difference in AFP between HBeAg^+^ and HBeAg^‐^ groups among cirrhotic patients, AFP was ~1.1‐fold higher in the HBeAg^‐^ group than that in the HBeAg^+^ group among patients without cirrhosis (*P* = .01) (Figure S2A‐D). Among HBeAg^+^ patients, the AFP was 87% lower in the antiviral group than that in the group without antiviral (*P* < .01) (Figure S2E‐H). Among patients without antiviral, serum AFP of cirrhotic group was ~5.2‐fold higher than that group without cirrhosis (*P* < .05) (Figure S2I‐L).

### 3.4 Diagnostic accuracy of serum biomarkers for detecting HBV‐HCC

AUC, sensitivity, specificity, LR+, and LR‐, as well as, optimal cut‐off for serum biomarkers (ALT, AST, AFP, and NLR) for diagnosing HBV‐HCC are shown in Table 3. AUC values for ALT, AST, AFP, and NLR were 0.533 (0.501‐0.565), 0.696 (0.666‐0.725), 0.84 (0.815‐0.862), and 0.684 (0.654‐0.713) with optimal cut‐off values of 43 IU/mL, 38 IU/mL, 7.21 ng/mL, and 2.61, respectively (Table 3). Among them, AFP showed the highest sensitivity (0.874) and specificity (0.744), while NLR individually showed the second highest sensitivity (0.807) and specificity (0.513).

**Table 3:** Diagnostic performances of serum biomarkers for detecting HBV‐HCC from CHB

Marker	Cut‐off value	AUC	Sensitivity (Sn)	Specificity (Sp)	Sn + Sp	LR+	LR−
ALT (IU/mL)	43	0.533 (0.501‐0.565)	0.616 (0.572‐0.658)	0.459 (0.413‐0.505)	1.075	1.14 (1‐1.3)	0.84 (0.7‐1)
AST (IU/mL)	38	0.696 (0.666‐0.725)	0.675 (0.632‐0.715)	0.641 (0.596‐0.685)	1.316	1.88 (1.6‐2.2)	0.51 (0.4‐0.6)
AFP (ng/mL)	7.21	0.84 (0.815‐0.862)	0.874 (0.842‐0.902)	0.744 (0.701‐0.784)	1.618	3.41 (2.9‐4)	0.17 (0.1‐0.2)
NLR	2.61	0.684 (0.654‐0.713)	0.807 (0.77‐0.84)	0.513 (0.466‐0.559)	1.32	1.66 (1.5‐1.8)	0.38 (0.3‐0.5)

Abbreviations AFP, alpha‐fetoprotein; ALT, alanine aminotransferase; AST, aspartate aminotransferase; AUC, area under the receiver operation characteristics curve; LR‐, negative likelihood ratio; LR+, positive likelihood ratio; NLR, neutrophil‐lymphocyte ratio.

Moreover, due to relatively lower sensitivity (0.616 or 0.675) and specificity (0.459 or 0.641) of ALT and AST, the diagnostic value of combined biomarkers was evaluated. The different combinations of these biomarkers were summarized in Table 4. Among all the alternative combinations of two biomarkers for diagnosis of HBV‐HCC, AFP combined with NLR was the highest AUC (0.865) with a significantly higher specificity (0.739) compared to other combinations of biomarkers. However, the suboptimal combination of AFP plus ALT or AST demonstrated the highest sensitivity (0.918 or 0.926) with relatively lower specificity (0.724 or 0.693). Considering the limited sensitivity or specificity of the combination of the two markers, we tried to utilize AFP combined with more biomarkers for detecting HBV‐HCC. Among all of the combinations with three biomarkers, AUC of the combination of AFP, ALT, and AST was 0.89, with the highest sensitive (0.888) and highest specificity (0.777) (Table 4). Furthermore, AFP plus ALT, AST, and NLR, showed the highest AUC (0.897) among all the three‐marker combinations, with sensitivity (0.877) and highest specificity (0.777). Based on these parameters and binary logistic regression analysis, the final equation was established: Y = −2.044 + 0.027 × AFP‐0.029 × ALT+0.036 × AST+0.309 × NLR (Table S1), which could be used for prediction of HBV‐HCC among CHB patients.

**Table 4:** Diagnostic performances of combinations of serum biomarkers for detecting HBV‐HCC from CHB

Marker	Cut‐off value	AUC	Sensitivity (Sn)	Specificity (Sp)	Sn + Sp	LR+	LR−
ALT + AST	0.466	0.794 (0.767‐0.819)	0.857 (0.823‐0.886)	0.616(0.57‐0.66)	1.473	2.23 (2‐2.5)	0.23 (0.2‐0.3)
ALT + NLR	0.486	0.687 (0.657‐0.716)	0.815 (0.778‐0.847)	0.502 (0.456‐0.548)	1.317	1.64 (1.5‐1.8)	0.37 (0.3‐0.5)
AST + NLR	0.47	0.722 (0.693‐0.75)	0.801 (0.763‐0.835)	0.558 (0.512‐0.604)	1.359	1.81 (1.6‐2)	0.36 (0.3‐0.4)
AFP + ALT	0.323	0.854 (0.83‐0.876)	0.918 (0.89‐0.941)	0.724 (0.68‐0.765)	1.642	3.33 (2.9‐3.9)	0.11 (0.08‐0.2)
AFP + AST	0.344	0.852 (0.828‐0.874)	0.926 (0.899‐0.947)	0.693 (0.648‐0.735)	1.619	3.02 (2.6‐3.5)	0.11 (0.08‐0.1)
AFP + NLR	0.358	0.865 (0.841‐0.886)	0.873 (0.841‐0.901)	0.739 (0.696‐0.779)	1.612	3.34 (2.9‐3.9)	0.17 (0.1‐0.2)
AFP + ALT + AST	0.36	0.89 (0.868‐0.909)	0.888 (0.857‐0.914)	0.777 (0.736‐0.815)	1.665	3.98 (3.3‐4.7)	0.14 (0.1‐0.2)
AFP + ALT + NLR	0.343	0.873 (0.85‐0.894)	0.863 (0.83‐0.892)	0.759 (0.717‐0.798)	1.622	3.58 (3‐4.2)	0.18 (0.1‐0.2)
AFP + AST + NLR	0.376	0.866 (0.843‐0.887)	0.885 (0.854‐0.912)	0.721 (0.677‐0.762)	1.606	3.17 (2.7‐3.7)	0.16 (0.1‐0.2)
AFP + ALT + AST + NLR	0.378	0.897 (0.879‐0.918)	0.877 (0.845‐0.905)	0.777 (0.735‐0.815)	1.654	3.93 (3.3‐4.7)	0.16 (0.1‐0.2)

Abbreviations AFP, alpha‐fetoprotein; ALT, alanine aminotransferase; AST, aspartate aminotransferase; AUC, area under the receiver operation characteristics curve; LR‐, negative likelihood ratio; LR+, positive likelihood ratio; NLR, neutrophil‐lymphocyte ratio.

**Figure 5:**
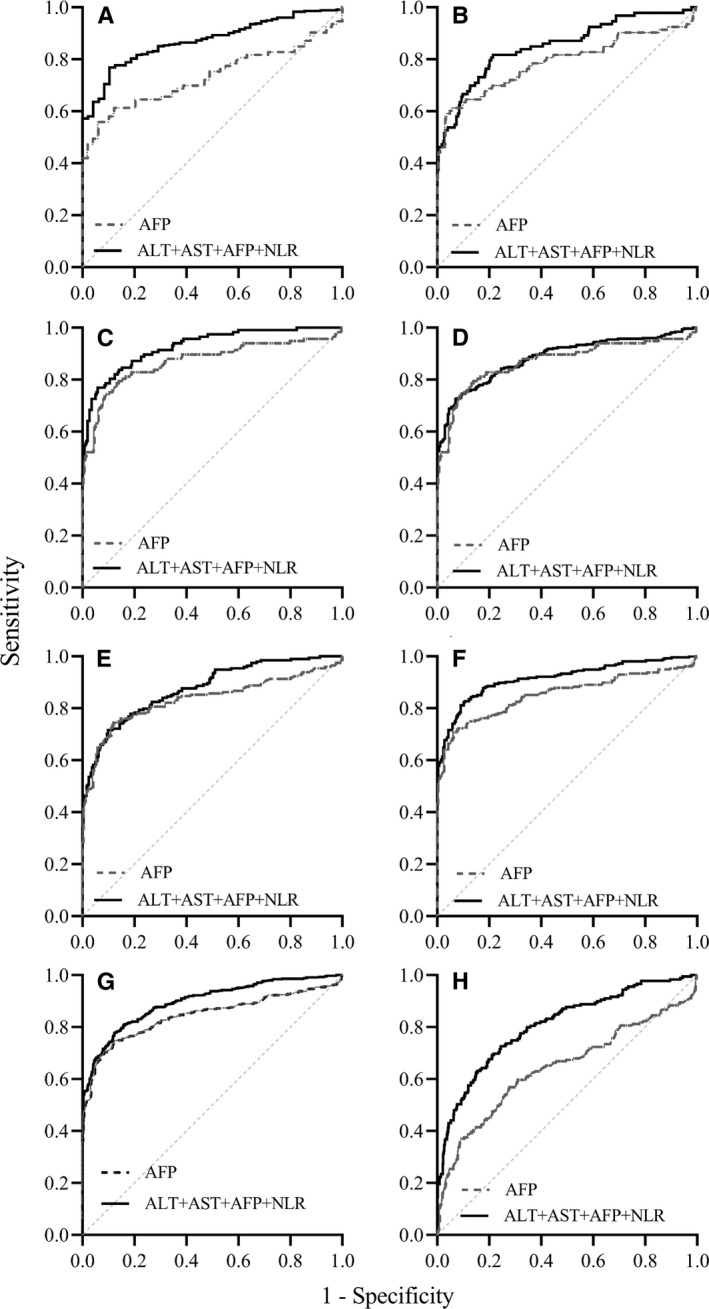
ROC curves of AFP and AFP combined ALT, AST, and NLR diagnosis for HBV‐HCC. ROC curves for AFP and AFP combined ALT, AST, and NLR in predicting the presence of HBV‐HCC in the 40‐60 years group (A), cirrhosis group (B), no‐cirrhosis group (C), HBeAg^+^ group (D), HBeAg^‐^ group (E), antiviral group (F), no‐antiviral group (G), AFP < 20 ng/mL group (H)

In the subgroup analysis, it was tested whether combination of multi‐indicator could provide better diagnostic value, compared with single one. After stratification of different age patients into subgroups, it was observed that the AUC value of AFP combined with ALT, AST, and NLR was 0.769 (95% CI: 0.727‐0.808), significantly better than AFP (*P* < .0001) alone or any joint indicators in 40‐60 years group. However, the diagnostic value of this combination in other age group did not achieve significance (Figure 5A). In the cirrhosis group, the AUC of AFP plus ALT, AST, and NLR was 0.873 (95% CI: 0.836‐0.903); with a sensitivity of 0.898 and a specificity of 0.769, better than any single use of AFP (*P* = .0052) or any indicator combination (Figure 5B). In the HBV‐HCC patients without cirrhosis group, the AUC of joint biomarker was 0.853 (95% CI: 0.82‐0.882); but AUC values did not reach significant difference between joint biomarkers and AFP (*P* = .0513) (Figure 5C). In the HBeAg^+^ group, the AUC of AFP combined with ALT, AST, and NLR was 0.928 (95% CI: 0.896‐0.952), superior to single use of AFP (*P* = .0145) (Figure 5D). Moreover, the AUC of uniting AFP with ALT, AST, and NLR was 0.885 (95% CI: 0.856‐0.91) in HBeAg^‐^ group, offering more optimal value than AFP (*P* = .0005) or other united indicators (Figure 5E). In addition, the AUC of AFP combined with ALT, AST, and NLR was 0.876 (95% CI: 0.841‐0.905) in antiviral group, which was higher than the single diagnostic value of AFP (*P* = .02) or any other combinations (Figure 5F). Among the no‐antiviral group, the AUC of the combined indicator was 0.915 (95% CI: 0.887‐0.938), provided higher diagnostic performance than AFP (*P* = .0002) (Figure 5G).

In the present study, there were 181 (35.49%) HBV‐HCC and 481 (94.31%) CHB patients whose serum AFP were lower than 20 ng/mL. Thus we further evaluated if the combined biomarkers could improve the diagnostic accuracy for HBV‐HCC. The AUC of AFP combine with ALT, AST, and NLR was 0.807 (95% CI 0.774‐0.836), showing diagnostic value was significantly better than other indicators and any other combinations (*P* < .001) (Figure 5H). Thus, AFP combination of ALT, AST, and NLR was superior to any single indicator or any combinations of each biomarkers, except in the CHB patients without cirrhosis group, and displayed a good diagnostic performance for detecting HBV‐HCC among almost all subgroups.

## 4 DISCUSSION

AFP is still routinely used in clinical practice as a conventional and relatively highly effective promising biomarker for surveillance and diagnosis for HCC over the past decades, despite small false positive or negative rates.^6^, ^9^ Although there are some biomarkers developed, such as des‐γ‐carboxy prothrombin (DCP),^18^ Glypican‐3 (GPC3),^19^ and alpha‐fetoprotein‐L3 (AFP‐L3),^20^ there is room to improve the diagnostic value for HCC, partially due to technique demand.^21^

Our current study observed that AFP accurately predicts the development of HBV‐HCC in CHB patients with a diagnostic accuracy that was superior to any single test, including ALT, AST, or NLR, suggesting that AFP still has a relatively high and stable diagnostic value for the HBV‐HCC in the Chinese population. Thus, the importance of AFP is not replaceable by other novel biomarkers yet, mainly due to economic/financial reason. This is supported by another study that reveals the combination of AFP plus ultrasonography is higher sensitivity than ultrasonography alone for early HCC detection in patients with cirrhosis 9 further suggesting that other combinations may possibly improve the diagnostic accuracy of HBV‐HCC.

Our univariate and multivariate analysis revealed that abnormal serum ALT, AST, AFP level, NLR and age, sex, cirrhosis, HBeAg^+^, or therapy were all individual independent risk factors for HBV‐HCC. Thus, regular monitoring of these biomarkers may be very useful in predicting the incidence of development of HBV‐HCC. This is consistent with others,^6^, ^22^, ^23^, ^24^ showing that ALT, AST, AFP, NLR, age, sex, cirrhosis, HBeAg^+^, and antiviral therapy probably are independent predictors for the development of HBV‐HCC. Thus, regular screening of these CHB patients could improve the success in predicting/discovering HBV‐HCC.

In our current study, AFP in HBV‐HCC patients receiving antiviral treatment was significantly lower than that in the no‐antiviral group, despite the observation that antiviral therapy did not alter serum AFP of CHB patients, suggesting that antiviral therapy will not be able to abolish completely the incidence of HBV‐HCC.

However, it has been reported that antiviral therapy improves the prognosis of HBV‐HCC patients,^25^ which is in line with our data, showing that antiviral therapy also reduced serum AFP. Interestingly, we also found that the AFP of the cirrhotic group was significantly higher than that of the noncirrhosis group among CHB patients, but AFP showed no significant difference between the cirrhosis and without cirrhosis groups in HBV‐HCC patients. Our explanation is that AFP is also regulated by persistence of HBV activation, sex, and tumor size. Our finding may reflect that AFP level is also regulated by other factors, such as HBV sustained activity, supported by the literature.^5^, ^6^, ^7^ Thus, AFP may not be used as the only indicator for predicting/ diagnosis of HBV‐HCC.

Through subgroup analysis, we found that antiviral therapy significantly reduced AFP in HBV‐HCC patients with HBeAg^+^, as well as, in the HBV‐HCC patients with cirrhosis compare to patients without these factors. Among no‐cirrhotic patients, the average level of AFP in the HBeAg^‐^ group was significantly higher than that in the HBeAg^+^ group, suggesting we should be wary about the possibility to develop HCC among these CHB patients. According to a large scale study in Greece, HCC risk remains increased in entecavir‐treated HBeAg^‐^ CHB patients with cirrhosis, particularly of older age, at least for the first 5 years of antiviral initiation.^26^ The data from our current study and the above results, indicate the advantage of regular monitoring of HCC among HBeAg^‐^ patients to improve the management of HBV‐HCC.^27^ It has also been reported that the majority of HBV‐HCC with low to normal HBV DNA, but without antiviral treatment were HBeAg^‐^, suggesting that antiviral therapy is critical even in these patients with normal HBV DNA.^28^ Taken together antiviral therapy can effectively reduce the incidence of HCC among HBeAg^‐^ CHB patients. It is necessary to be wary about the development of HCC among HBeAg^‐^ patients with abnormal AFP, because of the similar HCC incidence between HBeAg^‐^ and HBeAg^+^ patients.

Abnormal ALT is another predictor for HCC development 22, 29, 30 which is confirmed by our study, showing that elevated ALT was a key risk factor for CHB‐HCC. This is supported by other reports that long‐term abnormalities in serum levels of ALT are independent predictors for HCC.^22^, ^29^, ^31^ In addition, abnormal serum ALT levels after antiviral therapy are significant risk factor for the development of HCC, while rapidly normalized ALT might minimize the risk of HCC development in patients with HBV‐associated cirrhosis.^22^, ^32^ Therefore, sustained antiviral therapy is needed to reduce abnormal serum ALT level and decrease the risk of HBV‐HCC. Additionally, it is worth noting that the cut‐off value of ALT was 38 IU/mL in our current study which is lower than the Chinese national standard at 65 IU/L. Once AASLD standard (the ALT cut‐off 35 IU/L in male and 25 IU/L in female) was applied,^15^ abnormal ALT was not the risk factor for HBV‐HCC, suggesting that AASLD standard may not be the best to be used in the Chinese population.

A high level of AST correlates with a greater influx of HBV, that is associated with decreased overall survival in HCC patients.^33^ In the present study, we determined that abnormal AST level is an independent factor associated with HBV‐HCC. Although serum AST concentration was not significantly different between CHB patients with cirrhosis and without cirrhosis in the current study, the level of AST in HBV‐HCC patients was significantly higher in the patients with cirrhosis than those without cirrhosis. However, the literature shows that AST levels are significantly different between patients with CHB and those with cirrhosis, and AST could be an independently predictor in cirrhosis CHB patients.^33^ Such discrepancy between our data and the literature may be due to the difference in races, and/or difference in viral genotypes.

NLR, a simple biomarker, is available through routine clinically examination.^34^ In the present study, NLR was an independent biomarker in predicting HBV‐HCC within a larger cohort. In addition, the combination of NLR, ALT, AST, and AFP improves the diagnostic accuracy for HBV‐HCC. However, there was no benefit in diagnostic accuracy when any of these two biomarkers was applied. Interestingly, it is reported that the combination of NLR plus AFP is a reliable predictive biomarker in the diagnosis of HCC with any original etiology.^14^ Thus, our explanation for the difference between ours and other studies may be due to: we focused on HBV‐HCC only with large patients size, whereas theirs are any causes with relatively smaller patient size. We revealed that the significantly elevated NLR was observed in the HBV‐HCC compare to CHB only patients, but antiviral therapy significantly reduced NLR scores among HBV‐HCC patients. This is in line with others showing that elevated NLR correlated with higher risk of HCC 34 and antiviral treatment decreases the risk of HCC in patients with CHB.^35^, ^36^, ^37^ Furthermore, Pinato *et al* show that elevated NLR is associated with HCC stage, particularly the patients with a higher NLR level tending to have greater possibility of extrahepatic spread.^38^ However, our data suggest NLR has good diagnostic performance for detecting HBV‐HCC, which might indicated that NLR performs as potential critical role for diagnosis of different HBV‐HCC stages.

Although ALT, AST, and NLR could not be used alone as a surrogate biomarker for HBV‐HCC screening in CHB patients, our studies provided some evidence that adding ALT, AST, and NLR to AFP outperformed the diagnosis for HBV‐HCC compared with AFP alone or any other combinations. This observation is consistent with several other studies,^24^, ^27^, ^39^, ^40^ showing that both CHB and HBV‐HCC had a strong male (65.88% or 86.47%) preponderance. It is implied that due attention should be paid to elderly male CHB patients who have higher risk for HBV‐HCC. Moreover, AFP combine with ALT, AST, and NLR achieved significantly better diagnostic performance only in 40‐60 years patients for HBV‐HCC than AFP and any other combinations among different age patients, which might mean that application value of AFP is still not the best indicator in young or over 60‐year‐old patients. Apart from the noncirrhosis group, we found that the diagnostic value of AFP combined with ALT, AST, and NLR was superior to AFP only for HBV‐HCC among almost every subgroup. Such combination achieved the best diagnostic performance with highest AUC (0.807) with a sensitivity of 0.755 and specificity of 0.726 in AFP < 20 ng/mL group in our study. Interestingly, it is reported that the diagnostic value of AFP plus NLR provides optimal diagnostic value (AUC 0.762) in HCC.^14^ Our study was to clarify the diagnostic role of AFP combine with ALT, AST, and NLR among different subgroups. Thus, our study may have a better clinical practice reference value for diagnosis of HBV‐HCC.

The strength of our current study is large patients’ group size, adoption of stringent exclusion criteria and univariate and multivariable analysis to minimize possible bias. Data from the real world represent narrower spectrum of patients than those in other clinical trials, in which chronic liver disease patients with several etiology are often included.

There are some limitations in the current studies: first, selection bias could not be avoided in the retrospective study. Second, further research is needed to clarify the diagnostic value of elevated ALT, AST, and NLR in patients with different clinical stages of HBV‐HCC. Third, HBV‐HCC stage and HBV family history were not collected, which might influence the screening value of AFP and HCC stage in the above subgroups.

We cautiously draw a conclusion that elder age, male, elevated AFP, ALT, AST, and NLR, cirrhosis, HBeAg^+^, and no‐antiviral therapy were independent risk factors of HBV‐HCC. Additionally, AFP is still a relatively high sensitivity and specificity biomarker for surveillance and diagnosis for HBV‐HCC, AASLD standard of ALT may not be most appropriate for used in the Chinese population. The level of AST and NLR has certain diagnostic value in HBV‐HCC. The diagnostic performance of AFP combined with ALT, AST, and NLR for HBV‐HCC is significantly superior to single biomarkers or any other combinations among all patients and most subgroups, especially in normal serum AFP concentration group and 40‐60 years group, which will be performed in further clinical practice.

## 5 CONCLUSIONS

Elderliness, male, elevated ALT, AST, NLR, AFP, cirrhosis, HBeAg^+^, and no‐antiviral treatment were independent risk factors for HBV‐HCC. AASLD standard of ALT cut‐off value may not be used as ideally as an option in the Chinese population. Regular monitoring of HCC among HBeAg^‐^ patients with abnormal AFP to improve the management of HBV‐HCC is recommended. The diagnostic performance of AFP combined with ALT, AST, and NLR for HBV‐HCC was superior to single biomarker or any other combinations among these patients.

## CONFLICT OF INTEREST

The authors declared there is not conflicts of interest.

## AUTHOR CONTRIBUTIONS

Hui Wang was fully responsible for the conduct of this study. Hui Wang and Yezhou Ding designed the experiment. Hui Wang, Yezhou Ding, Kehui Liu and Yumin Xu coordinated the study. Yezhou Ding performed the majority of the experiment and drafted the manuscript. Shisan Bao & Hui Wang interpretation data and revised the manuscript. Clinical data collection was completed by Kehui Liu, Yumin Xu, Qingqing Zhao, Xiaogang Xiang, Lei Yan, Zhujun Cao, Chuanwu Zhu and Qing Xie. All authors have read and approved this final version of the manuscript.

## ETHICS STATEMENTS

The current study has been approved by the Human Ethic Committee, Ruijin Hospital, Shanghai Jiaotong University School of Medicine.

## Supporting information

Supplementary Material

## Data Availability

The data of retrospective study used to support the findings of this study are available from the corresponding author upon request.

## References

[1] El‐Serag HB . Epidemiology of viral hepatitis and hepatocellular carcinoma. Gastroenterology. 2012;142(6):1264‐1273. 22537432 10.1053/j.gastro.2011.12.061 PMC3338949

[2] Villanueva A . Hepatocellular carcinoma. N Engl J Med. 2019;380(15):1450‐1462. 30970190 10.1056/NEJMra1713263

[3] Forner A , Reig M , Bruix J . Hepatocellular carcinoma. Lancet. 2018;391(10127):1301‐1314. 29307467 10.1016/S0140-6736(18)30010-2

[4] Omata M , Cheng A‐L , Kokudo N , et al. Asia‐Pacific clinical practice guidelines on the management of hepatocellular carcinoma: a 2017 update. Hepatol Int. 2017;11(4):317‐370. 28620797 10.1007/s12072-017-9799-9 PMC5491694

[5] Kansagara D , Papak J , Pasha AS , et al. Screening for hepatocellular carcinoma in chronic liver disease. Ann Int Med. 2014;161(4):261‐269. 24934699 10.7326/M14-0558

[6] Trevisani F , Garuti F , Neri A . Alpha‐fetoprotein for diagnosis, prognosis, and transplant selection. Semin Liver Dis. 2019;39(2):163‐177. 30849784 10.1055/s-0039-1677768

[7] Atiq O , Tiro J , Yopp AC , et al. An assessment of benefits and harms of hepatocellular carcinoma surveillance in patients with cirrhosis. Hepatology. 2017;65(4):1196‐1205. 27775821 10.1002/hep.28895 PMC5659110

[8] Kanwal F , Singal AG . Surveillance for hepatocellular carcinoma: current best practice and future direction. Gastroenterology. 2019;157(1):54‐64. 30986389 10.1053/j.gastro.2019.02.049 PMC6636644

[9] Tzartzeva K , Obi J , Rich NE , et al. Surveillance imaging and alpha fetoprotein for early detection of hepatocellular carcinoma in patients with cirrhosis: a meta‐analysis. Gastroenterology. 2018;154(6):1706‐1718.e1. 29425931 10.1053/j.gastro.2018.01.064 PMC5927818

[10] Arzumanyan A , Reis HM , Feitelson MA . Pathogenic mechanisms in HBV‐ and HCV‐associated hepatocellular carcinoma. Nat Rev Cancer. 2013;13(2):123‐135. 23344543 10.1038/nrc3449

[11] Li BO , Zhou P , Liu Y , et al. Platelet‐to‐lymphocyte ratio in advanced Cancer: review and meta‐analysis. Clin Chim Acta. 2018;483:48‐56. 29678631 10.1016/j.cca.2018.04.023

[12] Templeton AJ , McNamara MG , Šeruga B , et al. Prognostic role of neutrophil‐to‐lymphocyte ratio in solid tumors: a systematic review and meta‐analysis. J Natl Cancer Inst. 2014;106(6):dju124. 24875653 10.1093/jnci/dju124

[13] Fest J , Ruiter R , Mulder M , et al. The systemic immune‐inflammation index is associated with an increased risk of incident cancer‐A population‐based cohort study. Int J Cancer. 2020;146(3):692‐698. 30924141 10.1002/ijc.32303 PMC6916270

[14] Hu J , Wang N , Yang Y , et al. Diagnostic value of alpha‐fetoprotein combined with neutrophil‐to‐lymphocyte ratio for hepatocellular carcinoma. BMC Gastroenterol. 2018;18(1):186. 30545306 10.1186/s12876-018-0908-6 PMC6293657

[15] Terrault NA , Lok ASF , McMahon BJ , et al. Update on prevention, diagnosis, and treatment of chronic hepatitis B: AASLD 2018 hepatitis B guidance. Hepatology. 2018;67(4):1560‐1599. 29405329 10.1002/hep.29800 PMC5975958

[16] Marrero JA , Kulik LM , Sirlin CB , et al. Diagnosis, staging, and management of hepatocellular carcinoma: 2018 practice guidance by the American Association for the Study of Liver Diseases. Hepatology. 2018;68(2):723‐750. 29624699 10.1002/hep.29913

[17] Mu R , Chen W , Pan B , et al. First definition of reference intervals of liver function tests in China: a large‐population‐based multi‐center study about healthy adults. PLoS One. 2013;8(9):e72916. 24058449 10.1371/journal.pone.0072916 PMC3772807

[18] Tanaka T , Taniguchi T , Sannomiya K , et al. Novel des‐gamma‐carboxy prothrombin in serum for the diagnosis of hepatocellular carcinoma. J Gastroenterol Hepatol. 2013;28(8):1348‐1355. 23432345 10.1111/jgh.12166

[19] Chen M , Li G , Yan J , et al. Reevaluation of glypican‐3 as a serological marker for hepatocellular carcinoma. Clin Chim Acta. 2013;423:105‐111. 23643963 10.1016/j.cca.2013.04.026

[20] Yi X , Yu S , Bao Y . Alpha‐fetoprotein‐L3 in hepatocellular carcinoma: a meta‐analysis. Clin Chim Acta. 2013;425:212‐220. 23954771 10.1016/j.cca.2013.08.005

[21] Chaiteerakij R , Addissie BD , Roberts LR . Update on biomarkers of hepatocellular carcinoma. Clin Gastroenterol Hepatol. 2015;13(2):237‐245. 24275343 10.1016/j.cgh.2013.10.038 PMC4032371

[22] Carr BI , Guerra V . A hepatocellular carcinoma aggressiveness index and its relationship to liver enzyme levels. Oncology. 2016;90(4):215‐220. 26974336 10.1159/000444394

[23] Hann H‐W , Wan S , Myers RE , et al. Comprehensive analysis of common serum liver enzymes as prospective predictors of hepatocellular carcinoma in HBV patients. PLoS One. 2012;7(10):e47687. 23112834 10.1371/journal.pone.0047687 PMC3480412

[24] Yuen M‐F , Tanaka Y , Fong D‐T , et al. Independent risk factors and predictive score for the development of hepatocellular carcinoma in chronic hepatitis B. J Hepatol. 2009;50(1):80‐88. 18977053 10.1016/j.jhep.2008.07.023

[25] Tada T , Kumada T , Toyoda H , et al. Long‐term prognosis of patients with hepatitis B infection: causes of death and utility of nucleos(t)ide analogue therapy. J Gastroenterol. 2015;50(7):795‐804. 25376770 10.1007/s00535-014-1011-6

[26] Papatheodoridis GV , Manolakopoulos S , Touloumi G , et al. Hepatocellular carcinoma risk in HBeAg‐negative chronic hepatitis B patients with or without cirrhosis treated with entecavir: HepNet.Greece cohort. Journal of viral hepatitis. 2015;22(2):120‐127. 25040685 10.1111/jvh.12283

[27] Papatheodoridis GV , Chan HL , Hansen BE , Janssen HL , Lampertico P . Risk of hepatocellular carcinoma in chronic hepatitis B: assessment and modification with current antiviral therapy. J Hepatol. 2015;62(4):956‐967. 25595883 10.1016/j.jhep.2015.01.002

[28] Zeng DW , Dong J , Zhang JM , Zhu YY , Jiang JJ , Liu YR . HBeAg‐negative chronic hepatitis patients should be monitored more strictly: a cross‐sectional retrospective study on antiviral treatment‐naive patients. J Med Virol. 2015;87(10):1682‐1688. 25965250 10.1002/jmv.24217

[29] Chen C , Lee W , Yang H , et al. Changes in serum levels of HBV DNA and alanine aminotransferase determine risk for hepatocellular carcinoma. Gastroenterology. 2011;141(4):1240‐1248. 21703214 10.1053/j.gastro.2011.06.036

[30] Kunutsor SK , Apekey TA , Seddoh D , Walley J . Liver enzymes and risk of all‐cause mortality in general populations: a systematic review and meta‐analysis. Int J Epidemiol. 2014;43(1):187‐201. 24585856 10.1093/ije/dyt192

[31] Suruki R , Hayashi K , Kusumoto K , et al. Alanine aminotransferase level as a predictor of hepatitis C virus‐associated hepatocellular carcinoma incidence in a community‐based population in Japan. Int J Cancer. 2006;119(1):192‐195. 16432841 10.1002/ijc.21796

[32] Kim EJ , Yeon JE , Kwon OS , et al. Rapid alanine aminotransferase normalization with entecavir and hepatocellular carcinoma in hepatitis B virus‐associated cirrhosis. Dig Dis Sci. 2017;62(3):808‐816. 28035553 10.1007/s10620-016-4431-8

[33] Lee KG , Seo YS , An H , et al. Usefulness of non‐invasive markers for predicting liver cirrhosis in patients with chronic hepatitis B. J Gastroenterol Hepatol. 2010;25(1):94‐100. 19793171 10.1111/j.1440-1746.2009.05953.x

[34] Fest J , Ruiter TR , Groot Koerkamp B , et al. The neutrophil‐to‐lymphocyte ratio is associated with mortality in the general population: The Rotterdam Study. Eur J Epidemiol. 2019;34(5):463‐470. 30569368 10.1007/s10654-018-0472-y PMC6456469

[35] Papatheodoridis GV , Sypsa V , Dalekos G , et al. Eight‐year survival in chronic hepatitis B patients under long‐term entecavir or tenofovir therapy is similar to the general population. J Hepatol. 2018;68(6):1129‐1236. 29427727 10.1016/j.jhep.2018.01.031

[36] Wang JP , Kao F‐Y , Wu C‐Y , et al. Nucleos(t)ide analogues associated with a reduced risk of hepatocellular carcinoma in hepatitis B patients: a population‐based cohort study. Cancer. 2015;121(9):1446‐1455. 25537961 10.1002/cncr.29159

[37] Wu C‐Y , Lin J‐T , Ho HJ , et al. Association of nucleos(t)ide analogue therapy with reduced risk of hepatocellular carcinoma in patients with chronic hepatitis B: a nationwide cohort study. Gastroenterology. 2014;147(1):143‐151. 24704525 10.1053/j.gastro.2014.03.048

[38] Pinato DJ , Stebbing J , Ishizuka M , et al. A novel and validated prognostic index in hepatocellular carcinoma: the inflammation based index (IBI). J Hepatol. 2012;57(5):1013‐1020. 22732513 10.1016/j.jhep.2012.06.022

[39] Jung KS , Kim SU , Ahn SH , et al. Risk assessment of hepatitis B virus‐related hepatocellular carcinoma development using liver stiffness measurement (FibroScan). Hepatology. 2011;53(3):885‐894. 21319193 10.1002/hep.24121

[40] Kulik L , El‐Serag HB . Epidemiology and management of hepatocellular carcinoma. Gastroenterology. 2019;156(2):477‐491. 30367835 10.1053/j.gastro.2018.08.065 PMC6340716

