# Supplementary material for: Combination of inflammatory score/liver function and AFP improves the diagnostic accuracy of HBV‐related hepatocellular carcinoma
Source: Cancer Med. 2020 Mar 9;9(9):3057–69. doi: 10.1002/cam4.2968 (PMC7196063; doi:10.1002/cam4.2968)
Supplement: Supplementary file 1 — Supplementary Material [file CAM4-9-3057-s001.docx]

**Supporting information**

**Tables of content:**

Supplementary Figure 1 (Fig. S1)

Supplementary Figure 2 (Fig. S2)

Supplementary Table 1 (Tab. S1)
Supplementary Table 2 (Tab. S2)

Supplementary Table 3 (Tab. S3)

Supplementary Table 4 (Tab. S4)
Supplementary Table 5 (Tab. S5)

Supplementary Table 6 (Tab. S6)

Supplementary Table 7 (Tab. S7)
Supplementary Table 8 (Tab. S8)

Supplementary Table 9 (Tab. S9)


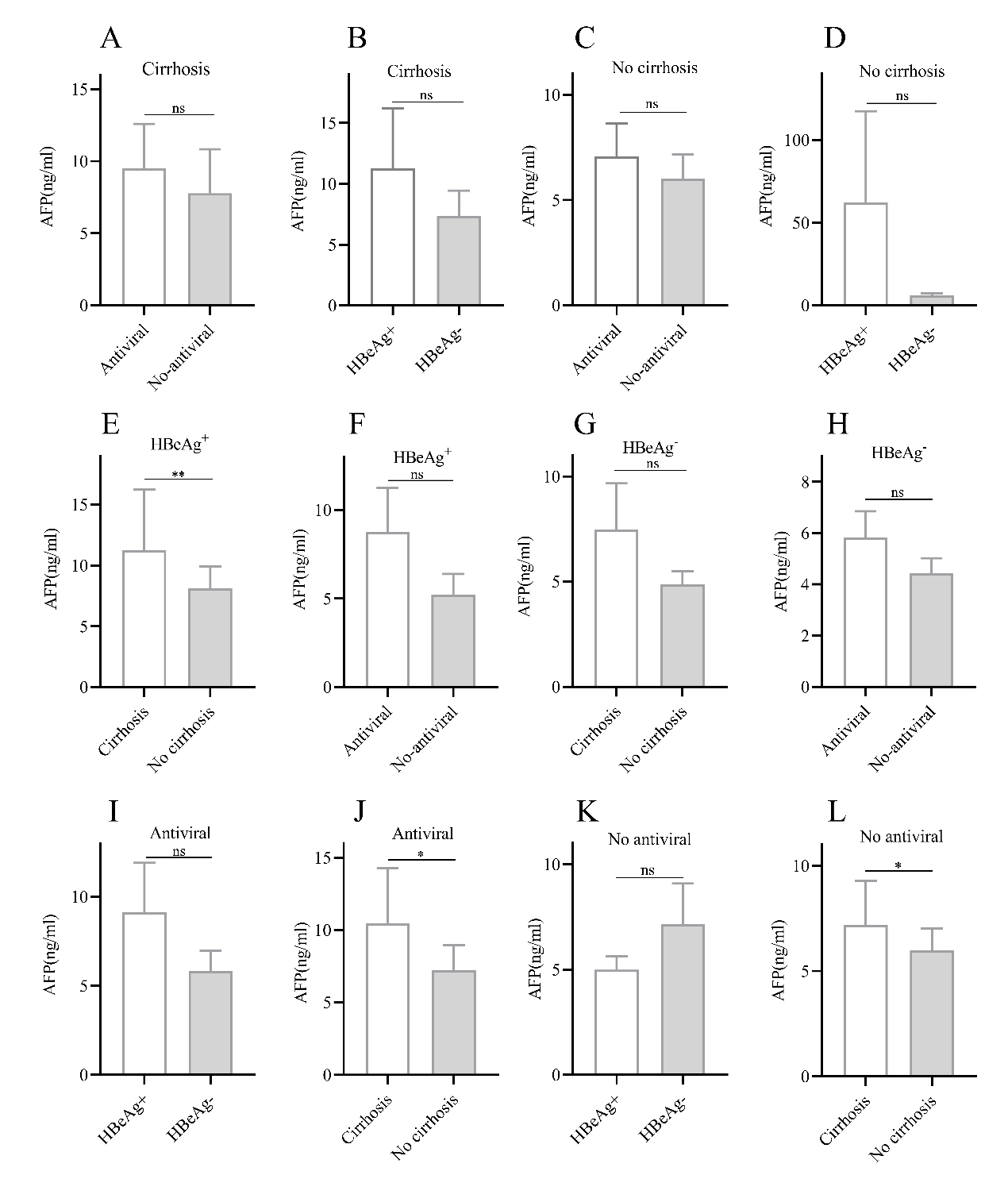


**Fig. S1. Analysis of serum AFP levels between different CHB subgroups**


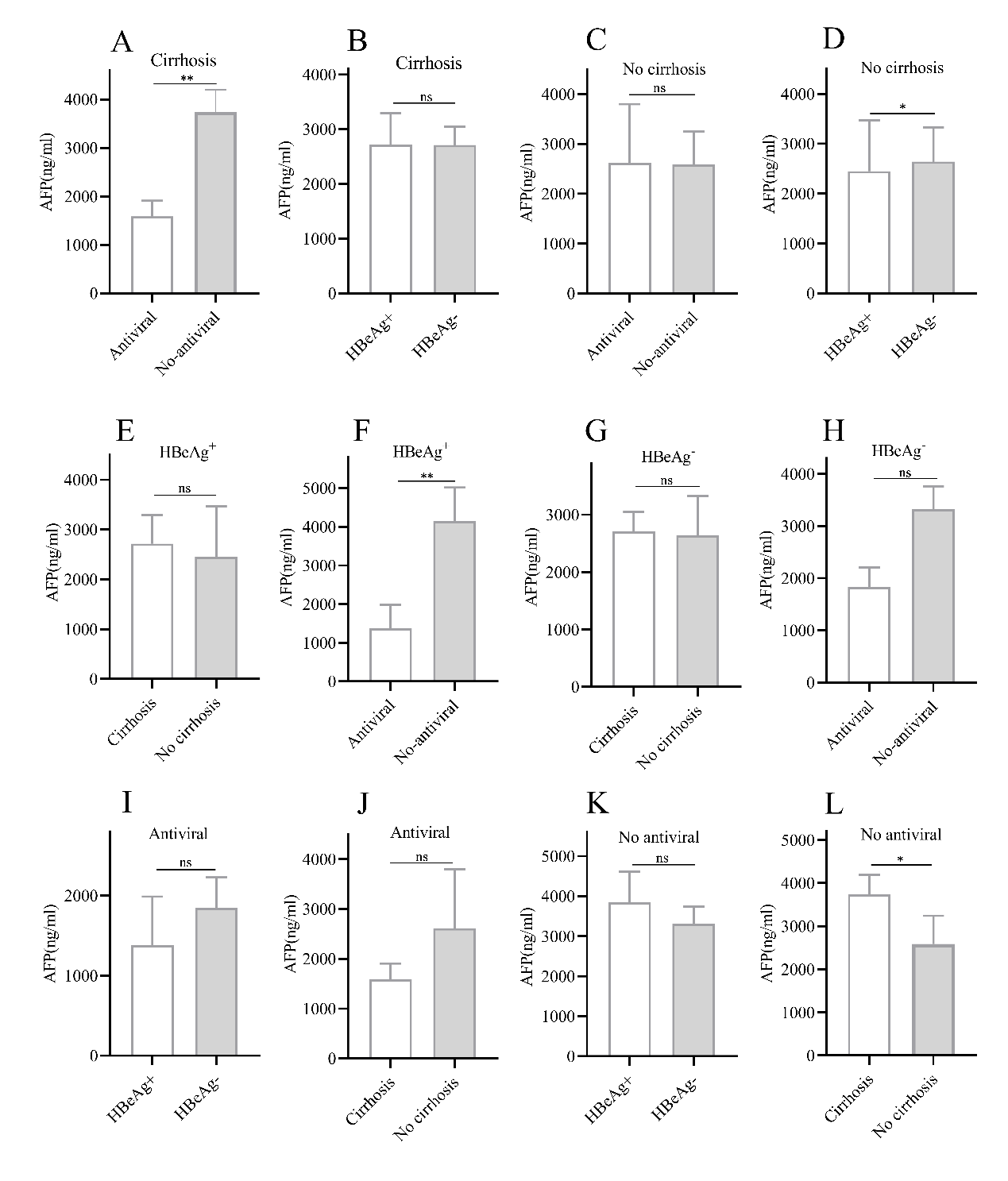


**Fig. S2.** **Analysis of serum AFP levels between different HBV-HCC subgroups**

**Tab. S1. Binary logistic regression to determine the best equation for probability prediction of HBV-HCC against CHB**

| Marker | Coefficient | Standard Error | *P* value |
| --- | --- | --- | --- |
| ALT (IU/L) | -0.029 | 0.004 | <0.05 |
| AST (IU/L) | 0.036 | 0.005 | <0.05 |
| AFP (ng/mL） | 0.027 | 0.005 | <0.05 |
| NLR | 0.309 | 0.054 | <0.05 |
| Constant | -2.044 | 0.193 | <0.05 |

Abbreviations: ALT, alanine aminotransferase; AST, aspartate aminotransferase; NLR, neutrophil / lymphocyte count; AFP, alpha-fetoprotein.

**Tab. S2. Diagnostic performances of combinations of serum biomarkers for detecting HBV-HCC from CHB in 40-60 years group**

| Marker | AUC | Sensitivity (Sn) | Specificity (Sp) | Sn + Sp | LR+ | LR- |
| --- | --- | --- | --- | --- | --- | --- |
| AFP | 0.627 | 0.284 (0.23-0.342) | 0.955 (0.913-0.98) | 1.239 | 6.27 (3.1-12.7) | 0.75 (0.7-0.8) |
| AFP+ALT | 0.633 | 0.317 (0.262-0.377) | 0.921 (0.871-0.956) | 1.238 | 4.01 (2.4-6.8) | 0.74 (0.7-0.8) |
| AFP+AST | 0.647 | 0.347 (0.29-0.407) | 0.91 (0.857-0.947) | 1.257 | 3.84 (2.3-6.3) | 0.72 (0.7-0.8) |
| AFP+NLR | 0.759 | 0.6 (0.538-0.659) | 0.851 (0.79-0.9) | 1.451 | 4.04 (2.8-5.8) | 0.47 (0.4-0.6) |
| AFP+ALT+AST | 0.652 | 0.392 (0.333-0.453) | 0.881 (0.824-0.925) | 1.273 | 3.3 (2.2-5.1) | 0.69 (0.6-0.8) |
| AFP+ALT+NLR | 0.759 | 0.626 (0.565-0.685) | 0.829 (0.764-0.881) | 1.455 | 3.65 (2.6-5.1) | 0.45 (0.4-0.5) |
| AFP+AST+NLR | 0.766 | 0.608 (0.546-0.667) | 0.851 (0.79-0.901) | 1.459 | 4.09 (2.8-5.9) | 0.46 (0.4-0.5) |
| AFP+ALT+AST+NLR | 0.769 | 0.581 (0.519-0.641) | 0.88 (0.822-0.924) | 1.461 | 4.84 (3.2-7.3) | 0.48 (0.4-0.6) |

Abbreviations: ALT, alanine aminotransferase; AST, aspartate aminotransferase; NLR, neutrophil / lymphocyte count; AFP, alpha-fetoprotein; AUC, area under the receiver operation characteristics curve; LR+, positive likelihood ratio; LR-, negative likelihood ratio.

**Tab. S3. Diagnostic performances of combinations of serum biomarkers for detecting HBV-HCC from CHB with cirrhosis**

| Marker | AUC | Sensitivity (Sn) | Specificity (Sp) | Sn + Sp | LR+ | LR- |
| --- | --- | --- | --- | --- | --- | --- |
| AFP | 0.813 | 0.878 (0.752-0.954) | 0.706 (0.656-0.752) | 1.584 | 2.98 (2.5-3.6) | 0.17 (0.08-0.4) |
| AFP+ALT | 0.811 | 0.857 (0.728-0.941) | 0.719 (0.67-0.765) | 1.576 | 3.06 (2.5-3.7) | 0.2 (0.1-0.4) |
| AFP+AST | 0.829 | 0.918 (0.804-0.977) | 0.669 (0.618-0.718) | 1.587 | 2.78 (2.3-3.3) | 0.12 (0.05-0.3) |
| AFP+NLR | 0.846 | 0.918 (0.804-0.977) | 0.69 (0.639-0.738) | 1.608 | 2.96 (2.5-3.5) | 0.12 (0.05-0.3) |
| AFP+ALT+AST | 0.855 | 0.878 (0.752-0.954) | 0.708 (0.658-0.755) | 1.586 | 3.01 (2.5-3.6) | 0.17 (0.08-0.4) |
| AFP+ALT+NLR | 0.848 | 0.939 (0.831-0.987) | 0.682 (0.63-0.73) | 1.621 | 2.95 (2.5-3.5) | 0.09 (0.03-0.3) |
| AFP+AST+NLR | 0.853 | 0.939 (0.831-0.987) | 0.679 (0.628-0.727) | 1.618 | 2.92 (2.5-3.5) | 0.09 (0.03-0.3) |
| AFP+ALT+AST+NLR | 0.873 | 0.898 (0.778-0.966) | 0.769 (0.722-0.812) | 1.667 | 3.89 (3.1-4.8) | 0.13 (0.06-0.3) |

Abbreviations: ALT, alanine aminotransferase; AST, aspartate aminotransferase; NLR, neutrophil / lymphocyte count; AFP, alpha-fetoprotein; AUC, area under the receiver operation characteristics curve; LR+, positive likelihood ratio; LR-, negative likelihood ratio.

**Tab. S4.** **Diagnostic performances of combinations of serum biomarkers for detecting HBV-HCC from CHB without cirrhosis**

| Marker | AUC | Sensitivity (Sn) | Specificity (Sp) | Sn + Sp | LR+ | LR- |
| --- | --- | --- | --- | --- | --- | --- |
| AFP | 0.79 | 0.942 (0.917-0.962) | 0.613 (0.506-0.712) | 1.555 | 2.43 (1.9-3.1) | 0.094 (0.06-0.1) |
| AFP+ALT | 0.834 | 0.934 (0.906-0.955) | 0.667 (0.561-0.761) | 1.601 | 2.8 (2.1-3.7) | 0.1 (0.07-0.1) |
| AFP+AST | 0.798 | 0.942 (0.917-0.962) | 0.613 (0.506-0.712) | 1.555 | 2.43 (1.9-3.1) | 0.094 (0.06-0.1) |
| AFP+NLR | 0.829 | 0.85 (0.814-0.882) | 0.742 (0.641-0.827) | 1.592 | 3.3 (2.3-4.7) | 0.2 (0.2-0.3) |
| AFP+ALT+AST | 0.846 | 0.92 (0.891-0.943) | 0.645 (0.539-0.742) | 1.565 | 2.59 (2-3.4) | 0.12 (0.09-0.2) |
| AFP+ALT+NLR | 0.845 | 0.877 (0.843-0.906) | 0.731 (0.629-0.818) | 1.608 | 3.26 (2.3-4.6) | 0.17 (0.1-0.2) |
| AFP+AST+NLR | 0.829 | 0.839 (0.802-0.872) | 0.753 (0.652-0.836) | 1.592 | 3.39 (2.4-4.8) | 0.21 (0.2-0.3) |
| AFP+ALT+AST+NLR | 0.853 | 0.786 (0.745-0.823) | 0.817 (0.724-0.89) | 1.603 | 4.3 (2.8-6.6) | 0.26 (0.2-0.3) |

Abbreviations: ALT, alanine aminotransferase; AST, aspartate aminotransferase; NLR, neutrophil / lymphocyte count; AFP, alpha-fetoprotein; AUC, area under the receiver operation characteristics curve; LR+, positive likelihood ratio; LR-, negative likelihood ratio.

**Tab. S5. Diagnostic performances of combinations of serum biomarkers for detecting HBV-HCC from CHB with HBeAg^+^**

| Marker | AUC | Sensitivity (Sn) | Specificity (Sp) | Sn + Sp | LR+ | LR- |
| --- | --- | --- | --- | --- | --- | --- |
| AFP | 0.87 | 0.861 (0.812-0.9) | 0.795 (0.71-0.864) | 1.656 | 4.19 (2.9-6) | 0.18 (0.1-0.2) |
| AFP+ALT | 0.871 | 0.903 (0.86-0.936) | 0.778 (0.692-0.849) | 1.681 | 4.06 (2.9-5.7) | 0.12 (0.08-0.2) |
| AFP+AST | 0.888 | 0.841 (0.791-0.883) | 0.829 (0.748-0.892) | 1.67 | 4.92 (3.3-7.4) | 0.18 (0.1-0.3) |
| AFP+NLR | 0.872 | 0.883 (0.838-0.92) | 0.727 (0.636-0.805) | 1.61 | 3.23 (2.4-4.4) | 0.16 (0.1-0.2) |
| AFP+ALT+AST | 0.918 | 0.841 (0.791-0.883) | 0.872 (0.797-0.926) | 1.713 | 6.56 (4.1-10.6) | 0.18 (0.1-0.2) |
| AFP+ALT+NLR | 0.877 | 0.949 (0.915-0.973) | 0.667 (0.574-0.751) | 1.616 | 2.85 (2.2-3.7) | 0.076 (0.04-0.1) |
| AFP+AST+NLR | 0.882 | 0.879 (0.833-0.917) | 0.743 (0.655-0.82) | 1.622 | 3.43 (2.5-4.7) | 0.16 (0.1-0.2) |
| AFP+ALT+AST+NLR | 0.928 | 0.942 (0.906-0.967) | 0.769 (0.682-0.842) | 1.711 | 4.08 (2.9-5.7) | 0.076 (0.05-0.1) |

Abbreviations: ALT, alanine aminotransferase; AST, aspartate aminotransferase; NLR, neutrophil / lymphocyte count; AFP, alpha-fetoprotein; AUC, area under the receiver operation characteristics curve; LR+, positive likelihood ratio; LR-, negative likelihood ratio.

**Tab. S6. Diagnostic performances of combinations of serum biomarkers for detecting HBV-HCC from CHB with HBeAg^-^**

| Marker | AUC | Sensitivity (Sn) | Specificity (Sp) | Sn + Sp | LR+ | LR- |
| --- | --- | --- | --- | --- | --- | --- |
| AFP | 0.835 | 0.893 (0.847-0.929) | 0.726 (0.675-0.773) | 1.619 | 3.26 (2.7-3.9) | 0.15 (0.1-0.2) |
| AFP+ALT | 0.836 | 0.901 (0.856-0.935) | 0.723 (0.672-0.77) | 1.624 | 3.25 (2.7-3.9) | 0.14 (0.09-0.2) |
| AFP+AST | 0.854 | 0.942 (0.905-0.968) | 0.679 (0.626-0.728) | 1.621 | 2.93 (2.5-3.4) | 0.085 (0.05-0.1) |
| AFP+NLR | 0.87 | 0.846 (0.794-0.889) | 0.773 (0.724-0.817) | 1.619 | 3.73 (3-4.6) | 0.2 (0.1-0.3) |
| AFP+ALT+AST | 0.879 | 0.934 (0.895-0.962) | 0.714 (0.663-0.762) | 1.648 | 3.27 (2.8-3.9) | 0.093 (0.06-0.1) |
| AFP+ALT+NLR | 0.874 | 0.858 (0.808-0.9) | 0.767 (0.718-0.812) | 1.625 | 3.69 (3-4.5) | 0.18 (0.1-0.3) |
| AFP+AST+NLR | 0.871 | 0.825 (0.771-0.871) | 0.801 (0.853-0.842) | 1.626 | 4.14 (3.3-5.2) | 0.22 (0.2-0.3) |
| AFP+ALT+AST+NLR | 0.885 | 0.913 (0.869-0.945) | 0.74 (0.689-0.787) | 1.653 | 3.51 (2.9-4.2) | 0.12 (0.08-0.2) |

Abbreviations: ALT, alanine aminotransferase; AST, aspartate aminotransferase; NLR, neutrophil / lymphocyte count; AFP, alpha-fetoprotein; AUC, area under the receiver operation characteristics curve; LR+, positive likelihood ratio; LR-, negative likelihood ratio.

**Tab. S7. Diagnostic performances of combinations of serum biomarkers for detecting HBV-HCC from CHB with antiviral**

| Marker | AUC | Sensitivity (Sn) | Specificity (Sp) | Sn + Sp | LR+ | LR- |
| --- | --- | --- | --- | --- | --- | --- |
| AFP | 0.829 | 0.881 (0.834-0.919) | 0.74 (0.672-0.8) | 1.621 | 3.39 (2.7-4.3) | 0.16 (0.1-0.2) |
| AFP+ALT | 0.845 | 0.91 (0.867-0.943) | 0.714 (0.646-0.776) | 1.624 | 3.18 (2.5-4) | 0.13 (0.08-0.2) |
| AFP+AST | 0.838 | 0.91 (0.867-0.943) | 0.709 (0.64-0.772) | 1.619 | 3.13 (2.5-3.9) | 0.13 (0.08-0.2) |
| AFP+NLR | 0.834 | 0.844 (0.792-0.887) | 0.706 (0.637-0.769) | 1.55 | 2.87 (2.3-3.6) | 0.22 (0.2-0.3) |
| AFP+ALT+AST | 0.878 | 0.881 (0.834-0.919) | 0.74 (0.672-0.8) | 1.621 | 3.39 (2.7-4.3) | 0.16 (0.1-0.2) |
| AFP+ALT+NLR | 0.84 | 0.91 (0.886-0.942) | 0.644 (0.573-0.712) | 1.554 | 2.56 (2.1-3.1) | 0.14 (0.09-0.2) |
| AFP+AST+NLR | 0.836 | 0.836 (0.783-0.88) | 0.717 (0.648-0.779) | 1.553 | 2.95 (2.3-3.7) | 0.23 (0.2-0.3) |
| AFP+ALT+AST+NLR | 0.876 | 0.901 (0.857-0.936) | 0.717 (0.648-0.779) | 1.618 | 3.18 (2.5-4) | 0.14 (0.09-0.2) |

Abbreviations: ALT, alanine aminotransferase; AST, aspartate aminotransferase; NLR, neutrophil / lymphocyte count; AFP, alpha-fetoprotein; AUC, area under the receiver operation characteristics curve; LR+, positive likelihood ratio; LR-, negative likelihood ratio.

**Tab. S8. Diagnostic performances of combinations of serum biomarkers for detecting HBV-HCC from CHB without antiviral**

| Marker | AUC | Sensitivity (Sn) | Specificity (Sp) | Sn + Sp | LR+ | LR- |
| --- | --- | --- | --- | --- | --- | --- |
| AFP | 0.849 | 0.938 (0.9-0.964) | 0.704 (0.644-0.759) | 1.642 | 3.17 (2.6-3.8) | 0.089 (0.05-0.1) |
| AFP+ALT | 0.864 | 0.926 (0.887-0.955) | 0.735 (0.677-0.788) | 1.661 | 3.5 (2.8-4.3) | 0.1 (0.07-0.2) |
| AFP+AST | 0.86 | 0.93 (0.891-0.958) | 0.716 (0.657-0.77) | 1.646 | 3.27 (2.7-4) | 0.098 (0.06-0.2) |
| AFP+NLR | 0.892 | 0.874 (0.827-0.912) | 0.795 (0.74-0.843) | 1.669 | 4.27 (3.3-5.5) | 0.16 (0.1-0.2) |
| AFP+ALT+AST | 0.899 | 0.918 (0.877-0.949) | 0.798 (0.743-0.845) | 1.716 | 4.54 (3.5-5.8) | 0.1 (0.07-0.2) |
| AFP+ALT+NLR | 0.902 | 0.882 (0.836-0.919) | 0.799 (0.745-0.847) | 1.681 | 4.39 (3.4-5.6) | 0.15 (0.1-0.2) |
| AFP+AST+NLR | 0.892 | 0.866 (0.818-0.905) | 0.799 (0.745-0.847) | 1.665 | 4.31 (3.4-5.5) | 0.17 (0.1-0.2) |
| AFP+ALT+AST+NLR | 0.915 | 0.909 (0.867-0.842) | 0.811 (0.757-0.857) | 1.72 | 4.81 (3.7-6.2) | 0.11 (0.08-0.2) |

Abbreviations: ALT, alanine aminotransferase; AST, aspartate aminotransferase; NLR, neutrophil / lymphocyte count; AFP, alpha-fetoprotein; AUC, area under the receiver operation characteristics curve; LR+, positive likelihood ratio; LR-, negative likelihood ratio.

**Tab. S9. Diagnostic performances of combinations of serum biomarkers for detecting HBV-HCC from CHB with AFP<** **20 ng/mL**

| Marker | AUC | Sensitivity (Sn) | Specificity (Sp) | Sn + Sp | LR+ | LR- |
| --- | --- | --- | --- | --- | --- | --- |
| AFP | 0.642 | 0.721 (0.679-0.761) | 0.569 (0.494-0.642) | 1.29 | 1.67 (1.4-2) | 0.49 (0.4-0.6) |
| AFP+ALT | 0.653 | 0.744 (0.703-0.783) | 0.569 (0.494-0.642) | 1.313 | 1.73 (1.4-2.1) | 0.45 (0.4-0.5) |
| AFP+AST | 0.661 | 0.707 (0.664-0.747) | 0.597 (0.521-0.669) | 1.304 | 1.75 (1.5-2.1) | 0.49 (0.4-0.6) |
| AFP+NLR | 0.731 | 0.785 (0.745-0.821) | 0.637 (0.562-0.707) | 1.422 | 2.16 (1.8-2.6) | 0.34 (0.3-0.4) |
| AFP+ALT+AST | 0.784 | 0.821 (0.784-0.854) | 0.635 (0.561-0.705) | 1.456 | 2.25 (1.8-2.7) | 0.28 (0.2-0.4) |
| AFP+ALT+NLR | 0.754 | 0.831 (0.794-0.863) | 0.603 (0.528-0.676) | 1.434 | 2.09 (1.7-2.5) | 0.28 (0.2-0.4) |
| AFP+AST+NLR | 0.734 | 0.799 (0.76-0.834) | 0.626 (0.55-0.697) | 1.425 | 2.14 (1.8-2.6) | 0.32 (0.3-0.4) |
| AFP+ALT+AST+NLR | 0.807 | 0.755 (0.714-0.793) | 0.726 (0.655-0.79) | 1.481 | 2.76 (2.2-3.5) | 0.34 (0.3-0.4) |

Abbreviations: ALT, alanine aminotransferase; AST, aspartate aminotransferase; NLR, neutrophil / lymphocyte count; AFP, alpha-fetoprotein; AUC, area under the receiver operation characteristics curve; LR+, positive likelihood ratio; LR-, negative likelihood ratio
